# Developing a Culturally Tailored Narrative Video for Skin Cancer Prevention Among Spanish-Speaking Hispanic Outdoor Workers: Co-Design Study

**DOI:** 10.2196/89986

**Published:** 2026-05-22

**Authors:** David Perez, Valeria Gómez, Dariana Sedeño-Delgado, Carlos Orellana García, Rosemary Sokas, Elissa Fairbrother, Jocelyn Apodaca Schlossberg, Amparo Caballero, Pilar Carrera, Otto Castillo Caldas, Melida Chacon, Lilia Correa-Selm, Nathaly Garcés Lenis, Geoffrey T Gibney, Nan Kyle Ficca, Ellen N Pritchett, Douglas Wilder, Edith Yañez, Rosa Yañez, Rebecca Young, Chiranjeev Dash, Roxanne Mirabal-Beltran, Alejandra Hurtado-de-Mendoza

**Affiliations:** 1 Georgetown University Medical Center, 2115 Wisconsin Avenue NW, Washington, DC, 20007, United States, 1 2026870100; 2 Baylor College of Medicine, Houston, TX, United States; 3 Impact Melanoma, Concord, MA, United States; 4 n/a, Los Angeles, CA, United States; 5 Universidad Autónoma de Madrid, Madrid, Madrid, Spain; 6 Honeywell Inc, Washington, DC, United States; 7 Spanish Catholic Center, Washington, DC, United States; 8 Department of Dermatology, USF Health, Tampa, FL, United States; 9 National Conservatory of Dramatic Arts, Washington, DC, United States; 10 Howard University Hospital, Washington, DC, United States; 11 Latinas in Construction, Washington, DC, United States; 12 LCR Electrical & Remodeling Corp, Davemport, FL, United States; 13 Farmworker Justice, Washington, DC, United States; 14 Georgetown University Berkley School of Nursing, Washington, DC, United States

**Keywords:** skin cancer prevention, Hispanic outdoor workers, cultural intervention, health communication, ultraviolet radiation, melanoma, community engagement, early detection

## Abstract

**Background:**

Hispanic individuals face a higher risk of skin cancer due to disproportionate occupational sun exposure; yet, culturally tailored resources for this demographic are scarce.

**Objective:**

The aim of this study was to codevelop a culturally tailored narrative video to enhance skin cancer prevention among Spanish-speaking Hispanic outdoor workers

**Methods:**

We partnered with the National Conservatory of Dramatic Arts, medical specialists, national skin cancer prevention organizations, occupational health experts, and Hispanic outdoor workers to create an educational video that promotes sun protection and skin cancer awareness and prevention among Hispanic outdoor workers and their families. Development was based on extensive formative research, health behavior models, and interactive script review by multistakeholder and community advisory boards.

**Results:**

The final result is a 27-minute video that follows Miguel, who is taking ownership of his father’s landscaping business as he is diagnosed with basal cell carcinoma. Throughout the video, Miguel and his wife, Sofía, learn about skin cancers, their warning signs, and steps for prevention, ultimately using this knowledge to protect and empower his employees and family. Key engagement strategies include cultural tailoring, story-driven learning, and visual modeling.

**Conclusions:**

This culturally and theoretically informed video represents a tool to increase knowledge and self-efficacy among Spanish-speaking Hispanic outdoor workers and their families, potentially reducing their skin cancer risk. Future research should evaluate the video’s acceptability and impact on enhancing awareness, knowledge, and sun-protective behaviors.

## Introduction

Skin cancer remains the most commonly diagnosed cancer in the United States [1]. In 2025, projections indicated that approximately 104,960 cases of melanoma and 5.4 million cases of nonmelanoma skin cancer—basal cell carcinoma (BCC) and squamous cell carcinoma (SCC)—would be diagnosed in the United States alone [2]. Exposure to solar UV radiation is the most common modifiable risk factor for skin cancer, making it largely preventable [3-7]. Outdoor workers have an increased risk of developing skin cancer as they are exposed to 8 times more UV radiation and are 60% more likely to develop skin cancer than indoor workers [8]. Hispanic individuals in the United States are overrepresented in outdoor jobs, including construction (53.9%) and landscaping or groundskeeping (48.1%), with men comprising the majority of this workforce [9-11]. Nationally, only 3.5% of construction laborers and 6.2% of groundskeeping workers identify as women. The incidence of both nonmelanoma and melanoma skin cancers has risen in Hispanic individuals over the past decades [12-15]. While non-Hispanic White individuals have a higher incidence of skin cancer, Hispanic patients of all races have worse health outcomes (eg, larger invasive tumors diagnosed at later stages) and higher mortality than non-Hispanic White patients [16-21]. Despite these disparities, most interventions and public health campaigns have been developed for and implemented among non-Hispanic White populations [22-24].

Sun protection behaviors are key to skin cancer prevention [25-28]. However, compared with non-Hispanic White individuals, Hispanic individuals are less likely to use sunscreen and wear protective clothing, sunglasses, and hats [28]. Even among Hispanic outdoor workers, who experience disproportionately high UV exposure, sun protection behaviors are suboptimal. In a population-based sample of Hispanic outdoor workers, 69% reported never or rarely wearing sunscreen while working outdoors [29]. Likewise, a study of Hispanic farmworkers found that only 9.2% used sunscreen and 11% used sunglasses [30]. Additionally, Hispanic individuals are less likely to perform skin self-examination (SSE), to be taught how to perform SSE, or to have a total skin examination conducted by a provider than non-Hispanic White individuals [28,31]. There is a critical need for culturally relevant materials among Hispanic populations, especially outdoor workers at high risk of sun exposure–related adverse health outcomes [25-29,32,33].

Hispanic individuals face multiple barriers to engaging in skin cancer prevention, including occupational (eg, workforce constraints), health care (eg, lack of access and socioeconomic factors), and psychosocial barriers (eg, knowledge, attitudes, and cultural factors). Studies have consistently shown very low awareness and knowledge about skin cancer and protective behaviors among this population, including outdoor workers [29,30,34,35]. In a study that had representation of different ethnic and racial groups, Hispanic individuals had the lowest skin cancer knowledge compared with other ethnic and racial groups but the highest level of interest to learn more about skin cancer, highlighting both an important gap and a key opportunity [26]. In addition, Hispanic individuals are more likely to have skin cancer misconceptions than non-Hispanic White individuals [28,32]. Common misconceptions and knowledge gaps include the beliefs that people with tanned to dark skin cannot develop skin cancer, that skin cancer would cause pain before diagnosis, that one cannot lower the risk of skin cancer, and that it is difficult to know which skin cancer prevention recommendations to follow [27,28,32,36]. There are also important knowledge gaps about sunscreen (eg, the meaning of sun protection factor) and how to use sunscreen properly. For example, a focus group study found that Hispanic men used sunscreen to cover up after sunburn to avoid pain [27].

Among Latino day laborers, sun-protective behaviors were more common when workers observed their supervisors practicing such behaviors [35]. Intention to engage in sun-protective behaviors has also been associated with normative beliefs such as how much peers engaged in sun-protective behaviors [37]. Some studies have shown that Hispanic individuals have negative attitudes about using sunscreen because they find it uncomfortable (eg, sticky and white cast) or believe that its use makes them feminine as a result of cultural beliefs such as *machismo*, which emphasizes strength and discourages behaviors perceived as vain [34]. On the other hand, *familismo*, a cultural value emphasizing the importance of and the need to take care of family, has been reported as a facilitator for Hispanic individuals to engage in sun-protective behavior [34]. Other social determinants of health, such as low education and low health literacy, have also been associated with lower engagement in sun-protective behaviors, lower perceived skin cancer risk, reluctance to perform SSEs out of fear of discovering skin cancer, and a greater number of sunburns [29,32,34,35].

Substantial evidence documents Hispanic individuals’ preference for interventions and education materials in Spanish that include plain language, visual aids, and a narrative format [13,18,37-39]. Narrative health education materials embed health information in the context of a character-driven story as opposed to traditional didactic materials, which rely primarily on facts and statistics [40,41]. Evidence suggests that narratives can be an effective strategy for cancer communication in Hispanic populations [38,41-43]. Video interventions have been shown to be more effective than written materials for sun protection and skin cancer education [44]. Studies have also documented Hispanic individuals’ preference for skin cancer video interventions over other types of interventions (eg, brochures) [26,36,45].

Additionally, Hispanic individuals are among the quickest-growing demographic in the United States, accounting for nearly 71% of the country’s growth between 2022 and 2023, and now comprising approximately 20% of the total population [46]. Despite national data showing that about 38% of US Hispanic adults primarily speak Spanish, prior research has repeatedly found limited availability of Spanish language dermatologic educational resources [45,47,48]. In response to this gap and the others previously identified, the goal of this study was to develop a culturally tailored narrative video intervention to promote skin cancer prevention among Spanish-speaking Hispanic men and women working in outdoor occupations. To date, and to our knowledge, this will be among the first video interventions specifically tailored to Spanish-speaking Hispanic outdoor workers. In this paper, we (1) describe how we used theoretical conceptual models and formative research to develop the script, (2) outline the script of the video, and (3) discuss the partnership and lessons learned among the research team, our advisory boards, and the National Conservatory of Dramatic Arts (NCDA) during script development.

## Methods

### Ethical Considerations

This study was reviewed by the Georgetown University Institutional Review Board and granted an exemption (Institutional Review Board ID: STUDY00007990) on August 23, 2024. In lieu of a traditional informed consent process, all community advisory board (CAB) and multistakeholder advisory board (MAB) members were required to sign a formal agreement form prior to participation, which outlined the roles and responsibilities of both the research team and the board members. The agreement detailed the research team's commitments, including ensuring fair participation opportunities, providing feedback on member engagement, and offering compensation. This form also detailed members' responsibilities, which included attending all 3 remote advisory board meetings via Zoom between January and March 2025, actively participating with cameras on and from distraction-free environments, and promptly notifying the research team of any scheduling conflicts or changes in contact information. Agreement forms were made available in both English and Spanish, according to each member's preference, to ensure accessibility and comprehension across membership. To protect member confidentiality, all members agreed, as part of their signed agreement form, to refrain from sharing names, addresses, phone numbers, or other identifying information about fellow members outside the group or on social media. All member contact information was stored securely in Box, a password-protected platform accessible only to members of the research team. Regarding compensation, advisory board members were offered US $150 per meeting attended, for a maximum total of US $450 per member across 3 meetings. MAB members were compensated via honorarium payments; prior to payment, members independently completed Georgetown University's online supplier registration process through the GU Supplier Registration portal, after which payment was directly deposited upon completion of their 3-meeting commitment. Notably, a few of the MAB members preferred to waive compensation and provided their expertise voluntarily to support the study's goals. CAB members were compensated via their choice of mailed check or direct deposit, also issued upon completion of their 3-meeting commitment. These payment methods were intentionally selected to prioritize convenience and accessibility for each respective group. Specifically, streamlined direct payment options requiring minimal paperwork were chosen for community board members specifically to reduce potential barriers associated with sharing sensitive personal or financial information.

### Theoretical Frameworks

The script was developed using an integrated approach grounded in 3 established behavioral theories: the Health Belief Model (HBM), the Theory of Planned Behavior (TPB), and Social Cognitive Theory (SCT). HBM explains health behaviors as a function of individuals’ perceptions of risk and the benefits of taking action [49]. HBM constructs addressed in the script include perceived susceptibility, perceived severity, perceived benefits, perceived barriers, and cues to action. TPB posits that behavior is shaped by intention, which in turn is influenced by attitudes, perceived subjective norms, and perceived behavioral control [50]. Tenants of TPB used in the script include attitudes toward behaviors, subjective norms, and perceived behavioral control. SCT emphasizes learning through observation and the role of self-efficacy and social support in behavior change [51]. SCT constructs integrated into the script include modeling, self-efficacy, social support, and outcome expectations.

We also included anticipatory emotions, as previous work, including our own, demonstrates that the inclusion of anticipatory emotions enhances the predictive capacity of behavioral health models [52-55]. Anticipatory emotions are emotions experienced in the present when thinking about future events (eg, feeling fear now when thinking about the possibility of a skin cancer diagnosis). Emotional appeals in health-related messages are effective in motivating behavior [52-55]. For instance, fear appeals can prime individuals to avoid harm by engaging in protective behaviors. However, they can also generate defensiveness when the message leads to higher perceived threat than self-efficacy [52-56]. Fear appeals should thus be accompanied with messages that increase self-efficacy and treatment effectiveness [52-55]. Cancer prevention and control interventions have mostly targeted negative emotions [52-55]. Nevertheless, mixed messages that sequentially introduce both negative and positive emotions toward a health behavior (vs only negative) generate lower defensiveness, increase response efficacy, and strengthen intentions to follow the health recommendation [52-55].

To complement these behavioral frameworks, the Narrative Immersion Model (NIM) was integrated to guide the story structure and improve message engagement [57]. The NIM builds on broader theories of narrative persuasion (eg, entertainment-education), which explain the mechanisms by which narratives engage audiences more effectively than purely informational messages [57]. Following the NIM, we incorporated structural elements shown to enhance the processes of immersion, including temporal structure, realism, perceived similarity, humor, and surprise, through an outcome narrative focused on the consequences of unprotected sun exposure [57].

### Formative Focus Group Data to Inform the Video Script

We leveraged our ongoing partnerships with community-based organizations (ie, Spanish Catholic Charities) to connect with and interview 25 outdoor workers across 3 focus groups [58]. Participants were predominantly male (22/25, 88%), 48% (12/25) had a middle school education or less, 52% (13/25) reported incomes ≤US $39,999, and 60% (15/25) were uninsured [58]. Results from these discussions gave the research team insight regarding existing skin cancer awareness, risk perception, risk reduction strategies, and barriers and facilitators for skin cancer prevention among Hispanic outdoor workers [58]. Findings from this study have been reported elsewhere [58]. The script also drew on existing literature and recommendations from organizations dedicated to skin cancer prevention [8,29,32,34,35,59,60].

### Methods to Develop and Obtain Feedback on Video Script

Following the analysis of our data, the research team assembled both a MAB and a CAB. The MAB consisted of members representing dermatologists, oncologists, family medicine clinicians, occupational health experts, national skin cancer prevention organizations, patient navigators, and Hispanic melanoma survivors. The CAB included Spanish-speaking outdoor workers from various outdoor sectors including agriculture, construction, and landscaping. Table 1 provides a description of the specific organizations and individuals on each board.

**Table 1.** Member characteristics.

Profession/expertise	Quantity
Multistakeholder advisory board	
Dermatologists	2
Oncologist	1
Family medicine clinician	1
IMPACT Melanoma and National Council on Skin Cancer Prevention	1
Latinas in Construction	1
Patient navigator	1
Hispanic melanoma survivor	1
Farmworker Justice	1
Professor of occupational and environmental medicine	1
Community advisory board	
Agricultural, construction, and landscaping representatives	3

We met with each advisory board separately for 2 hours across 2 sessions to review and gather suggestions for the script. The research team chose to establish 2 separate boards in order to create a space where CAB members could speak in Spanish and express their perspectives openly. For both cycles of input on the formative findings and script review, the CAB meeting was always held ahead of the MAB meeting. Recommendations from the CAB were then presented at each MAB meeting for consideration. The research team collaborated with the NCDA in Washington, DC, to develop the script and produce the video. NCDA members were present at every MAB meeting to engage with the board and ensure that suggestions were clearly understood before incorporating them in the script.

In the first meeting with our CAB, we presented the findings from our focus groups and asked about their thoughts regarding the results and content for the script. We subsequently met with our MAB and similarly discussed the focus group findings, the suggestions from our first CAB meeting, and gathered their recommendations. A first draft of the script was then developed by the NCDA. Upon obtaining the first draft of the script, the research team distributed the script to all board members for review.

Following a multiweek review period, we held a second meeting with our CAB to hear their thoughts on the script and gather their recommendations for refinement. We then met with our MAB to present the CAB’s suggestions and obtain the MAB’s recommendations. The research team subsequently consolidated and triangulated all suggestions before sending the script to the NCDA for final development.

After receiving the final version of the script, the research team translated the script into Spanish and consulted with a Spanish-speaking dermatologist on our MAB to ensure the medical terminology was accurate and consistent with dermatologic terms in Spanish.

## Results

### Findings From the Focus Group Formative Work

Previously unreported recommendations from participants addressed video content and dissemination, including presenting clear information about skin cancer and sun protection, featuring relatable characters, and sharing the video through workplaces, community networks, and social media (Table 2).

**Table 2.** Video suggestions from focus groups.

Themes	Responses
Video information	Clear, simple, and straightforward information. Use humor or entertaining elements to maintain engagement. Address what skin cancer is, its causes (sun, genetics, and environment), and symptoms. Explain treatment options and recovery. Provide guidance on sunscreen (SPF^a^ and recommended products), protective clothing, and hydration. Should be short or longer if engaging.
Characters	Include strong, relatable characters (eg, athletes who use sunscreen). Include families and children. Represent workers in relatable environments.
Visuals	Show real images of skin cancer. Show protective clothing (eg, color and material). Depict long-term consequences (childhood to adulthood).
Dissemination	Use social media, advertisements, and television. Distribute at worksites, in safety talks, and through flyers. Engage communities (eg, churches and Facebook groups). Share in schools, companies, and community centers. Encourage peer-to-peer awareness (eg, among coworkers).

a SPF: sun protection factor.

### Feedback From the CAB and the MAB

CAB recommendations focused on explaining the causes of skin cancer, signs to look for, and how to protect oneself. MAB feedback emphasized clear clinical details, dialogue about the most common skin cancer diagnoses, and concise and relevant information on melanoma and nonmelanoma skin cancer types. Both boards suggested incorporating family-centered activities, including culturally relevant foods and sports, and providing national and local resources. Table 3 shows a detailed breakdown of the specific recommendations from each advisory board and the resulting adaptations to the video script.

**Table 3.** Script recommendations from advisory boards (community advisory board and multistakeholder advisory board).

Category	CAB^a^ feedback	MAB^b^ feedback	Script adaptations
Skin cancer awareness	Extend conversation with the doctor to address questions, such as “What does this diagnosis mean?” and “What are the next steps?” Introduce the different types of skin cancers that exist for educational purposes^c^. Include images of how different types of skin cancer look^c^.	Have Miguel go to the doctor for a sore that was not healing, and display how the doctor performed a full skin examination. Clinical information should come from the physician scene in the video. Miguel’s diagnosis should be either basal or squamous cell carcinoma, as those are most common in outdoor workers. Introduce the different types of skin cancer that exist and briefly discuss melanoma^c^. Include images, information on warning signs, or ABCDEs of melanoma^c^^,d^. Include information about acral melanoma. Although it is not associated with UV radiation, it has a higher prevalence among Hispanic individuals.	Expanded the conversation between the physician and Miguel to include more information about his diagnosis, treatment, and timeline for recovery. In this scene, the physician also briefly explains the different types of skin cancers that exist and shows images and ABCDEs. Miguel is diagnosed with basal cell carcinoma.
Skin cancer risk perception	Include a scene toward the end where Miguel is talking to his staff about their risk of UV exposure and his experience with a skin cancer diagnosis.	Describe federal regulations regarding occupational skin cancer risk.	Included a scene toward the end where Miguel calls for a company meeting. During the scene, Miguel shares his diagnosis, addresses common misconceptions about skin cancer risk, and discusses the steps he plans to take to protect his workers.
Skin cancer risk reduction strategies	Display how to conduct self-checks and encourage the audience to check with their physician for concerning moles and other skin irregularities suspected to be skin cancer^c^.	Emphasize the importance of regular screenings and early detection, not just when the disease is already advanced. Emphasize prevention throughout the video. Display how to conduct self-checks^c^. Discuss and emphasize the different types of sunscreen (cream, spray, roll-on, etc). Introduce risk reduction that can be implemented across all sectors (construction, landscaping, and agriculture). Do not mention SunGuard (Rit) as it has not been recommended by the American Academy of Dermatology.	Emphasized the importance of conducting self-checks as a risk reduction strategy. Embedded message focusing on early detection and prevention throughout the scenes. Explored different forms of sunscreen based on preference.
Barriers and facilitators for risk reduction behaviors	Include or introduce cultural role models practicing sun-safe behaviors, such as sports athletes, to motivate male outdoor workers. Show the steps Miguel takes as a company owner to protect his workers (eg, providing them sunscreen) from UV exposure. Ensure that the physician reassures Miguel after the diagnosis so that he feels uplifted and empowered to know that he can fully recover after treatment^c^.	Include a patient navigator to reiterate the information discussed in the video. Have one of Miguel’s workers ask whether skin cancer hurts, and address this in the video. Explain how to use clothing as a form of protection and highlight differences between clothing that protects from heat vs UV radiation. Introduce UPF^e^ clothing and the impact of different clothing colors on sun absorption. Include a list of national and local resources at the end of the video. Include a scene or image at the end summarizing the key points or messages of the video. Emphasize that sun protection is easy and affordable^c^.	Included a scene toward the end where Miguel calls for a company meeting. During the scene, Miguel shares his diagnosis, addresses common misconceptions about skin cancer risk, and discusses the steps he plans to take to protect his workers. Embedded positive and uplifting messaging throughout the video, including Miguel’s actions to protect himself, his workers, and his family from UV radiation. Included extended discussion in the work meeting scene about UPF clothing, color of clothing, and other appropriate protection strategies. Included a list of local and national resources in the end credits.
Cultural aspects	Change food and drinks from the initial script to traditional foods. Change the way Miguel receives the biopsy results to a phone call. Hispanic individuals do not usually check email or patient portal^c^. Emphasize the importance of protecting one’s family^c^. Change baseball game to soccer^c^.	Include a family or friend soccer game at the end to include teaching point in action. Do not include expressions conveying “machismo” in the video as it may distract from the main messages. Extend scenes with family gatherings^c^. Change the way Miguel receives the biopsy results to a phone call. Hispanic individuals do not usually check email or patient portal^c^.	Removed pizza from script and replaced it with traditional foods such as pupusas and carne asada. Adapted the news of biopsy results to a phone call instead of email. Emphasized the message of family unity and support throughout the script. Added a scene at the end where Miguel and his family are having a picnic and children are playing soccer. This scene serves to summarize the main points discussed throughout the video.

a CAB: community advisory board.

b MAB: multistakeholder advisory board.

c Feedback that was provided by both the CAB and MAB.

d ABCDE: Asymmetry, Border, Color, Diameter, Evolution.

e UPF: ultraviolet protection factor.

### Video Description

The 27-minute video consists of 8 scenes, each designed to address specific behavioral constructs. The video narrates the story of Miguel, a landscape worker who is transitioning to lead his father’s landscaping company when he is diagnosed with BCC. The cancer is detected early and he undergoes a minor surgery, but the diagnosis raises concerns about the potential skin cancer risks for his family and employees. In his visits with the doctor, Miguel and his wife, Sofía, learn about different types of skin cancers (BCC, SCC, and melanoma), skin cancer signs, the importance of early detection, and key sun-protective behaviors. Following these visits, Miguel and Sofía share the information with their relatives and implement new sun safety practices at their workplace. Throughout the video, we target key Integrated Behavioral Model theoretical constructs as well as the barriers and facilitators we found in our formative work [58]. For instance, the video dispels common misconceptions (eg, skin cancer cannot occur in people with darker skin) and addresses knowledge gaps (eg, most efficient protective clothing). The video also models behaviors (eg, how to properly apply sunscreen) to increase self-efficacy and illustrates how subjective norms change throughout the narrative, from many coworkers being reticent about sunscreen use to then valuing and using sunscreen by the end. Finally, we included superficial cultural tailoring, such as having Hispanic actors, filming the video in Spanish, and including traditional Hispanic foods and popular sports such as soccer. Deep structure tailoring, addressing key core values and beliefs, included appealing to *familismo* as a core motivation to engage in sun-protective behavior and in portraying family support. Table 4 provides a detailed description of the video by scene, outlining the content and the theoretical constructs addressed in each segment.

**Table 4.** Description of video by scene.

Scenes: Description/messages	Conceptual model constructs addressed
*Scene 1. Work*: The film begins at a landscape work site where Miguel is working with his brother and coworkers. 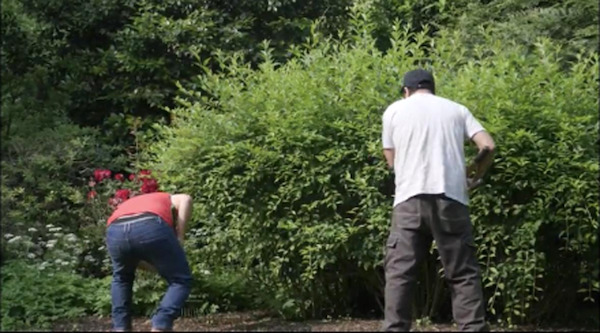	Health belief model: *Perceived susceptibility*: Presents common misconceptions (eg, Hispanics do not need sun protection).
*Scene 2. Diagnosis*: While preparing for a family dinner, Miguel receives a phone call from the doctor confirming positive biopsy results for skin cancer. His wife notices his concern and asks him about it. 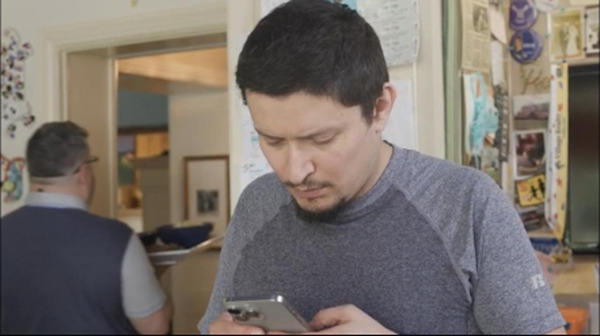	Health belief model: *Perceived severity*: Miguel recognizes the seriousness of his diagnosis, feeling emotional concern not only for himself but also for the impact it may have on his family. Social cognitive theory: *Self-efficacy*: After informing Sofía and engaging her support, Miguel gains confidence in his ability to navigate the treatment process and face the challenges ahead. *Emotional appeal:* The cancer diagnosis causes fear, but telling Sofía results in reassurance, balancing the negative emotion with support.
*Scene 3. Doctor consultation*: Miguel and Sofía discuss his diagnosis of BCC with the doctor and next steps for treatment. 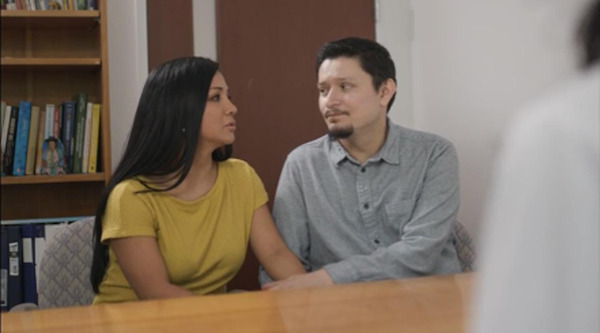	Health belief model: *Perceived susceptibility*: The doctor explains that skin cancer can develop across decades. *Perceived severity*: The doctor explains prognoses and treatment of detected vs undetected melanocytic and nonmelanocytic skin cancer. *Perceived barriers*: *machismo* and denial. *Perceived benefits*: Early detection leads to simpler treatment. *Cues to action*: The doctor reinforces skin checks, warning signs (eg, ABCDEs^a^ of melanoma), and follow-up care. Theory of planned behavior: *Attitudes toward behavior*: Miguel and Sofía develop a positive outlook toward treatment and prevention. Social cognitive theory: *Outcome expectations*: Clear explanation of treatment outcomes and benefits of early action. *Social support*: Sofía’s presence and encouragement highlight the importance of family support in health decisions. *Emotional appeal:* Revisits fear by emphasizing the seriousness of cancer but also introduces self-efficacy and reduces defensiveness as the doctor explains treatment options.
*Scene 4. Daydream*: Miguel daydreams of the moment he told his son he did not need to wear sunscreen while working in the garden. Miguel remembers how his father would tell him that there was no need for protection. Miguel decides to be proactive and teach his employees and children about sun protection. 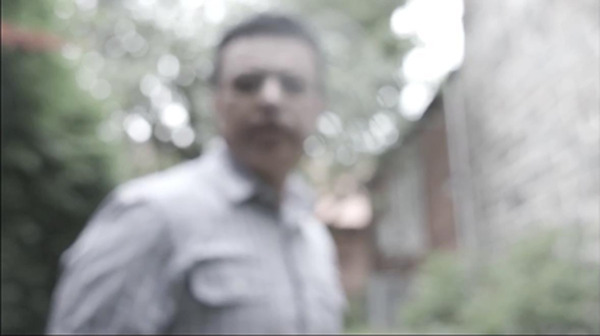	Health belief model: *Perceived susceptibility*: Miguel imagines his son facing the same diagnosis, personalizing the risk and recognizing intergenerational vulnerability. *Perceived benefits*: Miguel sees value in prevention for his family and workers. *Perceived barriers*: Miguel acknowledges misinformation he once believed and passed on as barriers. *Cues to action*: Dream functions as a cue and prompts Miguel to reconsider past behaviors. Theory of planned behavior: *Attitude toward behavior*: Previously viewed sunscreen as unnecessary; dream reflects a shift in attitude. *Perceived behavioral control*: Miguel believes that he can implement changes with Sofía’s help and support from the doctor. Social cognitive theory: *Modeling*: Dream reveals how Miguel internalized negative attitudes about sun-protective behaviors from his father, subsequently teaching them to his children. *Outcome expectations*: Miguel understands that behavior changes can protect his son and workers in the future. *Emotional appeal*: Regret and fear about past inaction are offset by Miguel’s drive to educate his employees and family.
*Scene 5. Family dinner*: Miguel shares his diagnosis with his family. 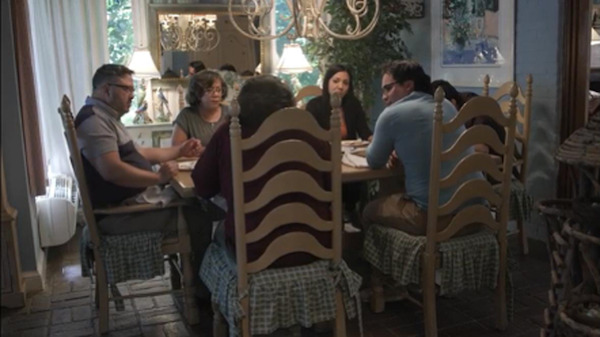	Health belief model: *Perceived severity*: Skin cancer diagnosis presents emotional concern in family. Theory of planned behavior: *Subjective norms*: Expectation of family involvement. Social cognitive theory: *Outcome expectations*: Family expects that a united approach will lead to better outcomes. *Self-efficacy:* Miguel gains confidence through family support. *Emotional appeal*: Despite the worry about his diagnosis, there is a shift toward hope as they prepare to face his cancer together.
*Scene 6. Doctors’ office*: Miguel asks the doctor about strategies to protect his team and his family. 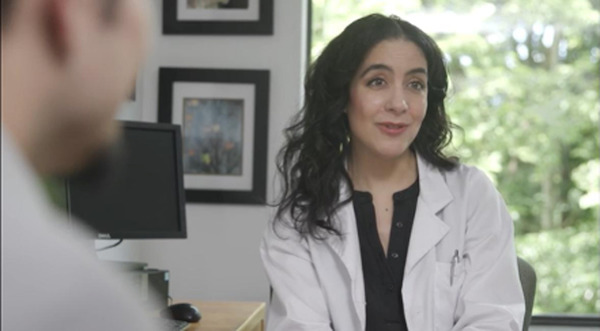	Health belief model: *Perceived susceptibility*: Miguel now acknowledges risk, not just for himself but for his workers and family members. *Perceived severity*: The experience of treatment reinforces that skin cancer is serious but manageable if addressed early. Theory of planned behavior: *Attitudes toward behavior*: Now associate prevention and follow-up with empowerment and responsibility, rather than fear. Social cognitive theory: *Modeling*: Miguel becomes a model of transformation. *Outcome expectations*: Early action and education can prevent future harm to others. *Social support*: The doctor provides emotional and informational support to sustain Miguel’s motivation. *Response efficacy*: The doctor explains the effectiveness of the recommended response. *Emotional appeal*: Miguel turns uncertainty into empowerment.
*Scene 7. Work place*: Miguel and Sofía share his diagnosis with his employees along with recommendations for sun-protective behaviors while dispelling common myths. 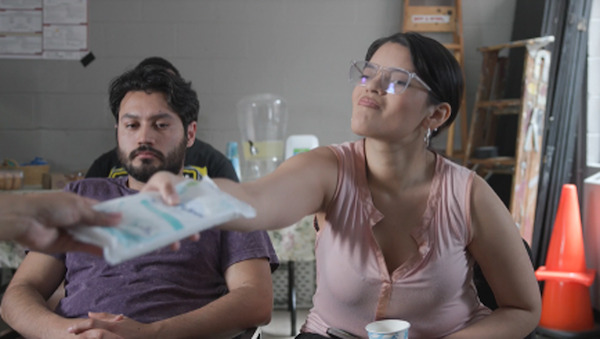	Health belief model: *Perceived susceptibility*: Miguel explains that outdoor workers are at higher risk for skin cancer due to daily UV exposure, even in cold or cloudy weather. *Perceived severity*: Miguel discloses his skin cancer diagnosis and underscores the potential seriousness of ignoring symptoms or misconceptions. *Perceived benefits*: The team learns how sun protection (eg, UPF^b^ clothing, sunscreen, etc) can reduce long-term risk. *Perceived barriers*: Workers express common concerns (eg, sunscreen discomfort, environmental barriers, disbelief regarding the need for sun protection, etc), but Miguel and Sofía address each with practical solutions. *Cues to action*: Miguel’s testimony and new workplace protocols (gear, sunscreen access, and flyers) act as strong motivators. Theory of planned behavior: *Attitudes toward behavior*: Initially negative or dismissive attitudes are challenged and reframed through lived experience and evidence. *Perceived behavioral control*: Workers are provided with tools (sunscreen, hats, and educational materials) to make protective behaviors more feasible. *Subjective norms*: Although workers voice negative attitudes initially, attitudes change and sun-protective behaviors are valued at the end. Social cognitive theory: *Modeling*: Miguel teaches his employees how they can protect themselves; Sofía also provides flyers for family engagement. *Outcome expectations*: Preventive behaviors are linked to staying healthy and being present for loved ones. *Self-efficacy*: Miguel and Sofía empower workers with concrete steps and tools. *Emotional appeal*: Balances concern about UV risks among employees and their families with collective self-efficacy.
*Scene 8. Family/friends gathering*: Miguel and Sofía share the importance of sun protection with friends working in other industries and reinforce the importance of sun-protective behaviors with their kids who are playing soccer. 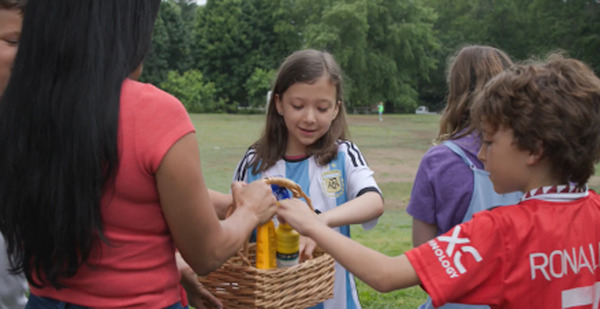	Health belief model: *Perceived susceptibility*: Miguel’s story motivates Arturo (a construction worker) to check a mole; the risk is now seen as real and personally relevant by peers. *Perceived severity*: Recognition that long-term sun exposure without protection has serious consequences, even if symptoms seem minor. *Perceived benefits*: Articulated through praise from Teresa and another outdoor worker. *Perceived barriers*: Addressed with practical tools (eg, visors, neck flaps, multiple sunscreen formulations, etc) and culturally relevant role models (eg, renowned soccer athletes) to normalize sun protection and reduce stigma. *Self-efficacy*: Everyone now has the knowledge, tools, and social support to feel confident in protecting themselves and their families. Theory of planned behavior: *Attitudes toward behavior*: Positive reinforcement of sun safety behaviors as part of being a good parent, worker, and friend. Social cognitive theory: Multiple generations model protective behavior in a communal setting. *Outcome expectations*: Reinforced via testimonials (eg, Arturo’s early detection, new workplace changes, etc). *Emotional appeal*: Joy and pride from seeing family and peers engage in sun protection. Fear has been overcome.

a ABCDEs: Asymmetry, Border, Color, Diameter, Evolution.

b UPF: ultraviolet protection factor.

## Discussion

### Principal Findings

The development of our educational video aimed at promoting sun protection among Hispanic outdoor workers and their families incorporated lessons learned from prior community outreach materials as well as guidance from workers, community leaders, and other stakeholders. In contrast to past approaches, our video uses entertainment-education, using a culturally contextualized narrative and visual demonstrations to impact knowledge, risk perception, and self-efficacy. Entertainment-education is unique among other approaches in that it delivers health messages through engaging stories that increase identification and reduce resistance to achieve lasting changes in attitudes and behaviors, demonstrating effectiveness in diverse contexts with empirical evidence and practical experiences [61,62].

Collaboration with national organizations, medical specialists, occupational health professionals, and community advisory members was crucial to the development of the video. Advisory boards built from personal experiences along with professional knowledge helped make the content practical and culturally sensitive. As reported by Cargo and Mercer [63], keeping community voices at the forefront of decision-making minimized technical or distant messaging, ensuring that behavior change strategies mirror real-life situations. Iterative review between the research and production teams was vital to preserving scientific accuracy while supporting the development of scenes that would resonate with our target audience. For instance, feedback from the CAB led to more scenes illustrating family members practicing sun safety and a soccer game to showcase healthy habits in culturally meaningful settings. These suggestions were consistent with findings from Perez et al [58], which highlight the value of a family-centric approach as well as appealing to the use of sunscreen by cultural role models (eg, soccer players). Feedback from the MAB, which was always informed by CAB suggestions, similarly resulted in expanding the doctor-patient conversation to clarify the differences in risks between BCC, SCC, and melanoma, as well as their prognosis and treatment timelines. It also prompted the inclusion of brief information regarding acral lentiginous melanoma because of its higher incidence among Hispanic White individuals than among non-Hispanic White individuals, despite being unrelated to occupational UV exposure [64,65]. Each scene thus reflects the integration of advisory board recommendations with theoretical concepts, behavioral goals, and cultural adaptations. This ensured that the video remained connected to both research and community priorities in concise, clear, and meaningful ways. Several challenges emerged from this development process, revealing key lessons about creating a culturally tailored, theory-driven video for health behavior change.

### Embedding Health Messages Within Storylines

Producing an engaging video required balancing essential health information about skin cancer prevention with a culturally meaningful storyline, while keeping the length short enough for dissemination across various platforms. This meant trimming scenes, limiting transitions, and reducing extraneous lines, all of which required iterative reviews of the script, as well as weighing which elements were essential for educational impact versus narrative appeal. This demonstrated the value of not only developing the scenes based on specific health messaging but also discussing potential time distributions for each scene with the production team at the onset of script development.

### Ensuring Gender Relevance

Creating a gender-inclusive video required balancing culturally tailored content for a Hispanic outdoor workforce, which is predominantly men, while carefully integrating the representation of female workers [66]. To achieve this, Sofía acts as a primary educator in the video. She specifically educates female outdoor workers in scene 7 and addresses their specific concerns about workplace challenges to sun protection. Outside the workplace, Sofía demonstrates family-focused sun protection by showing how protective habits can fit into everyday life. In addition to discussing practical strategies for outdoor workers and their families, she models them in many ways (eg, sharing a basket of sunscreen with the children playing soccer in scene 8). Through these interactions, Sofía emphasizes the key role women have in promoting sun-safe behaviors both at work and home. Importantly, these strategies were shaped by female viewpoints. This included insights from 2 female members of the CAB and collaborations with female-led groups, such as Latinas in Construction, ensuring that women’s voices were consistently included throughout the process.

### Balancing Technical Language With Acting Techniques

As reported by Hurtado-de-Mendoza et al [67], scenes discussing medical information require strict adherence to the script. When making this video, we had to ensure that the actors delivered their lines exactly as written, as even minor paraphrasing, which is common in filmmaking, could misrepresent important health information. For example, describing a new skin change as a “pimple,” which suggests something temporary and harmless, instead of as a “bump,” which can indicate a concerning change, is critical. Translating these terms into Spanish with precision in ways that are understandable added a layer of complexity, but it was facilitated by our MAB, which included a Spanish-speaking dermatologist. This process highlighted the additional time and effort it may take to film content that is both medically accurate and engaging, especially when there are time and budget constraints.

### Coordinating Multiple Advisory Boards

Managing input from 2 advisory boards required careful planning and execution. Scheduling across time zones and differing availability among clinicians, researchers, organizations, and community members often resulted in longer timelines. However, creating a distinct space in which community members could use their native language allowed us to build rapport and elicit conversations that yielded genuine and thorough suggestions. This approach underscored the importance of structuring engagement in a way that welcomes differing perspectives without diminishing the contributions of any group.

### Addressing Behavioral Complexity in Prevention Interventions

Prevention-based messaging for modifiable behavioral risks such as UV exposure, which are shaped by a range of daily behaviors, contexts, and environments, must anticipate multiple pathways of action and resistance. This includes but is not limited to addressing various considerations such as why outdoor workers may avoid sunscreen, the range of protective methods available, and the questions participants may have about each option. Messaging must not only provide guidance across multiple scenarios, but it must also recognize and support the agency associated with behavioral risks to encourage choices that directly influence risk.

### Keeping the Video as Neutral and Timeless as Possible

Throughout our board discussions, we remained intentional about creating a video that would be clear for diverse audiences and remain accurate and relevant over time. A topic we extensively discussed, for example, was whether to show images of skin cancers. While we illustrated the ABCDEs (Asymmetry, Border, Color, Diameter, Evolution) of melanoma and described how skin cancers may look through dialogue, we ultimately chose not to include specific images as we recognized that Hispanic individuals have a wide range of skin tones. Thus, a particular image of how skin cancer looks in one person may not reflect how cancer appears on someone else. Showing such images could mislead viewers, leading them to think that a skin change that looks different from the example shown is harmless. Additional information and resources can be included as complementary materials to the video in future interventions. Furthermore, although questions about the role of government in preventing occupational skin cancer were raised by various collaborators, the prospects for action were unclear, and the decision was made to focus on empowering outdoor workers directly, as we recognize that not all employers provide resources regarding skin cancer awareness and prevention [68,69]. Therefore, all information in the video was limited to factual content, avoiding anything that could be misunderstood or become outdated. This logic was helpful as we considered other recommendations, and it also supported our aim of creating a video focused primarily on prevention among outdoor employees.

### Strengths and Limitations

This project was strengthened by its theory-based and community-involved process. Most psychoeducation interventions follow a traditional didactic approach. Our video is innovative because it uses a narrative communication approach to convey health information through a story developed in collaboration with our advisory boards to ensure that the narrative resonates with the target population. The use of constructs from the HBM, TPB, and SCT ensured that the script addressed multiple determinants of behavior change, while input obtained through separate advisory boards allowed for open dialogue within each board and minimized the influence of professional hierarchies. Additional strength was provided by our partnership with a range of stakeholders, including dermatologists, occupational health experts, and representatives from various national organizations involved in skin cancer prevention. Outdoor workers consulted on the project also represented diverse geographic areas, including the District of Columbia, Maryland, Virginia, and Florida, increasing the cultural and contextual applicability of the script. The video format also provides practical benefits for sharing and scalability. Its visual and narrative style can overcome literacy barriers, demonstrate protective behaviors, and stress socially supported practices in ways that traditional print or educational materials cannot. Recognizing that the video was produced in Spanish and not all Hispanic outdoor workers may speak Spanish, however, we also created a version with English subtitles. In addition to helping it remain accessible in a lens that still resonates with non-Spanish speakers, given the overarching cultural values and experiences it is grounded in, the video can be broadly shared via various channels, including workplace training, community health clinics, and online platforms. Despite these strengths, the script was developed for a particular demographic and may require modification for use in alternate populations or settings beyond landscaping and construction. Additionally, although the CAB included representatives who were small business owners, we recognize that this level of representation was not sufficient to ensure comprehensive incorporation of employer-level perspectives. As a result, important organizational perspectives relevant to workplace implementation may not have been fully captured. Furthermore, while the development process was informed by theory and stakeholder feedback, the impact of the intervention on behavior change has yet to be evaluated and should be the focus of future research.

### Future Research Recommendations

Additional research needs to be conducted to assess the acceptability and efficacy of our video on sun protection behaviors, psychosocial (eg, knowledge and self-efficacy) outcomes, and process evaluation outcomes. In addition to obtaining preliminary feedback from outdoor workers, future approaches could include a randomized controlled trial in which the video is compared with a control arm (eg, traditional didactic video or fact sheet) with longitudinal follow-up. This may also include the development and piloting of complementary tools or resources on sun protection, followed by studies comparing their efficacy to the video. Subsequent studies could also explore knowledge and behavioral changes not only among workers but also among their families to provide insight into the broader effects of the video, recognizing that its plot and call to action were guided by the principle of *familismo*. Pending these findings, future work should also consider adapting the video into English for non–Spanish-speaking outdoor workers and engaging a broader range of employers to ensure that workplace perspectives and implementation insights are fully incorporated. This could include a multilevel intervention addressing individual behaviors and organizational supports for sun protection, including workplace facilitators and barriers, integrating the video into workplace wellness and safety programs, input on acceptability, and alignment with existing training.

### Conclusions

By combining formative research with input from both CAB and MAB, we developed a theory-driven, contextually relevant intervention that is responsive to the gaps reported in the work of others as well as our own. The resulting video is both a reflection of and response to the occupational realities faced by Hispanic outdoor workers, demonstrating how codeveloped interventions can bridge the gap between evidence and experience. Rather than imposing predefined messages, our approach relied on active listening and iterative content-tailoring, representing a departure from passive information transfer. We present this as an innovative approach in an effort to increase engagement, comprehension, and sun-protective behaviors among this population relative to traditional didactic methods. Based on the active participation from the workers who were involved in the video’s development, the video appears to have strong potential for effective implementation in real-world settings. More broadly, this project illustrates how integrating community participation with theory-based frameworks can enhance translation from research to practice, foster engagement among underserved populations, and promote health equity through culturally resonant prevention strategies.

## Abbreviations

ABCDEs: Asymmetry, Border, Color, Diameter, Evolution
BCC: basal cell carcinoma
CAB: community advisory board
HBM: Health Belief Model
MAB: multistakeholder advisory board
NCDA: National Conservatory of Dramatic Arts
NIM: Narrative Immersion Model
SCC: squamous cell carcinoma
SCT: Social Cognitive Theory
SPF: sun protective factor
SSE: skin self-examination
TPB: Theory of Planned Behavior
UV: ultraviolet
